# Clinical presentation and management of snakebite envenoming in northern Ghana

**DOI:** 10.1371/journal.pntd.0013820

**Published:** 2025-12-12

**Authors:** Leslie Mawuli Aglanu, John Humphrey Amuasi, Ibrahim Kwaku Duah, Melvin Katey Agbogbatey, Jonathan Steinhorst, Austin Gideon Adobasom-Anane, Zakari Bukari, Tia Joseph Azabu, Benno Kreuels, David Griffith Lalloo, Ymkje Stienstra

**Affiliations:** 1 University Medical Centre Groningen, Department of Internal Medicine/Infectious Diseases, University of Groningen, Groningen, The Netherlands; 2 Global Health and Infectious Diseases Research Group, Kumasi Centre for Collaborative Research in Tropical Medicine, Kumasi, Ghana; 3 Research Group Global One Health, Section for Implementation Research, Bernhard Nocht Institute for Tropical Medicine, Hamburg, Germany; 4 Department of Global Health, School of Public Health, Kwame Nkrumah University of Science and Technology, Kumasi, Ghana; 5 Division for Tropical Medicine, Department of Medicine, University Medical Centre Hamburg-Eppendorf, Hamburg, Germany; 6 College of Nursing and Midwifery, North East Region, Nalerigu, Ghana; 7 Research Group Neglected Diseases and Envenoming, Section for Implementation Research, Bernhard Nocht Institute for Tropical Medicine, Hamburg, Germany; 8 Wa Municipal Hospital, Upper West Region, Wa, Ghana; 9 Baptist Medical Centre, North East Region, Nalerigu, Ghana; 10 Department of Medicine, School of Medicine, Kamuzu University of Health Sciences, Blantyre, Malawi; 11 Centre for Snakebite Research and Interventions, Liverpool School of Tropical Medicine, Liverpool, United Kingdom; Instituto Butantan, BRAZIL

## Abstract

**Background:**

Snakebite envenoming is among the top five emergency health conditions in northern Ghana. Among the four genera of snake species classified to be of highest medical importance, species with haemotoxic venom are responsible for about 90% of all snakebite case presentations in the region. However, there is a dearth of clinical data on signs and symptoms of envenoming, treatment practices and health outcomes. We examined the signs and symptoms of envenoming and clinical management practices at referral hospitals in northern Ghana.

**Methods:**

Medical records of patients reporting on account of snakebite between 2016 and 2020 at the Wa Municipal Hospital in the Upper West region and the Baptist Medical Centre in the North East region of Ghana were reviewed. Demographic characteristics, patients’ clinical data and management practices were analysed and evaluated taking into consideration the national standard treatment guideline.

**Results:**

A total of 2,684 records of patients reporting on account of snakebite were accessed at both health facilities over the five-year period. 91% of the patients were admitted to the ward. Swelling, severe pain and bleeding were the most common clinical signs upon presentation. A total of 1,670 (64.7%) of all the patients tested had at least one abnormal blood clotting result suggesting haemotoxicity. Antivenom was administered to 84.3% of the patients. Antibiotics were administered to 70.5% with amoxicillin with clavulanic acid, flucloxacillin and metronidazole accounting for 59.2% of all antibiotics administered. The recorded case-fatality rate was 1.9%.

**Conclusion:**

The annual hospital attendance rate on account of snakebite to the Wa Municipal Hospital and the Baptist Medical Centre is estimated at 55 persons per 100,000 population per year. Mortality was low, with antivenom available to most of the patients. More evidence is needed on the indication and dosing of antivenom and to improve appropriate ancillary care.

## Author summary

Snakebite envenoming is among the top five emergency health conditions in northern Ghana. However, there is a dearth of clinical data on signs and symptoms of envenoming, treatment practices and health outcomes. We reviewed medical data of 2,684 patients reporting on account of snakebite to two referral health facilities in snakebite endemic regions in Ghana. The majority of patients presented with signs and symptoms suggesting haemotoxic envenoming with a two-days median length of hospitalisation. Over 80% of the patients were treated with antivenom and the mortality rate was below 2%. More context specific evidence is needed to guide the indication and dosing of antivenom and to reduce the delivery of unnecessary and ineffective ancillary care.

## Introduction

Limited access to adequately staffed and resourced healthcare facilities compounds the treatment challenges of common medical emergencies such as snakebite envenoming (SBE) in many rural tropical regions. Available estimates suggest Sub-Saharan Africa is among the regions with the highest snakebite morbidity and mortality rates [1–3]. Bites from different species in the region are associated with distinct clinical signs and symptoms, ranging from mild local effects such as pain and swelling to severe local tissue damage necessitating surgery and life-threatening systemic envenoming, including severe hypotension, neurotoxicity or even death [4,5]. Apart from the physical burden, SBE can also lead to severe psychosocial and economic consequences for patients and their families [6–8].

Upon presentation to a health facility, an accurate diagnosis and determination of the severity of envenomation is essential in guiding appropriate treatment. However, the lack of diagnostic tools for early detection and monitoring of envenoming presents a major impediment to effective management [9–11]. Healthcare workers in many rural tropical regions rely on patients’ history, knowledge on local snake species and basic laboratory investigations such as the 20-minute whole blood clotting test (20WBCT) to diagnose SBE and to estimate the most likely species responsible for the bite [12–14]. The complexity of clinical care for snakebite, combined with the limited attention given to SBE management in medical curricula suggest treatment may often be inadequate in many snakebite-endemic countries [15–17].

Although there are a number of medically important snake species in Ghana including the Black-necked Spitting Cobra (*Naja nigricollis)*, Puff Adder (*Bitis arietans)*, West African Green Mamba (*Dendroaspis viridis*), the Black Mamba (*Dendroaspis polylepis*) and the Night Adder (*Causus maculatus*), the West African Carpet Viper (*Echis ocellatus*) (S1 Appendix) is thought to cause about 90% of snakebite envenomings in northern Ghana [6,18–20]. Based on our assessment of health facility reported data between 2015–2020 from the District Health Information Management System of Ghana (DHIMS), the hospital attendance rate on account of snakebite in northern Ghana is estimated to be 76 persons per 100,000 population per year. In a community-based study involving household units in northern Ghana, a lifetime snakebite prevalence of 6% and a case-fatality ratio of about 3% was estimated [18]. Many studies have highlighted that a high number of rural snakebite patients do not visit or may not reach the formal healthcare system for treatment [21–23]. In a recent community survey in Ghana, about 40% of patients did not seek allopathic treatment from a health facility [6]. Access to well-resourced healthcare facilities, availability of antivenom and cost of treatment are among the factors influencing hospital attendance rate [24–26].

In Ghana, few studies on clinical manifestations and treatment practices in hospital settings have been undertaken to generate evidence needed to guide interventions at the health system level [19,20,27,28]. In this study, we present a comprehensive description of clinical features and management practices among patients presenting to the hospital on account of snakebite in northern Ghana by retrospectively evaluating patient medical records from two major health facilities.

## Methods

### Ethics statement

Ethics approval was obtained from the Ghana Health Service Ethics Review Committee (GHS-ERC010/03/20), the Medical Ethical Committee of the University Medical Centre Groningen, The Netherlands (NL-LTc201900047) and the Christian Health Association of Ghana Research Unit (CHAG-IRB 03062021). Access and permission to use the data was obtained from the medical directors of the participating hospitals.

### Study design

**Fig 1:**
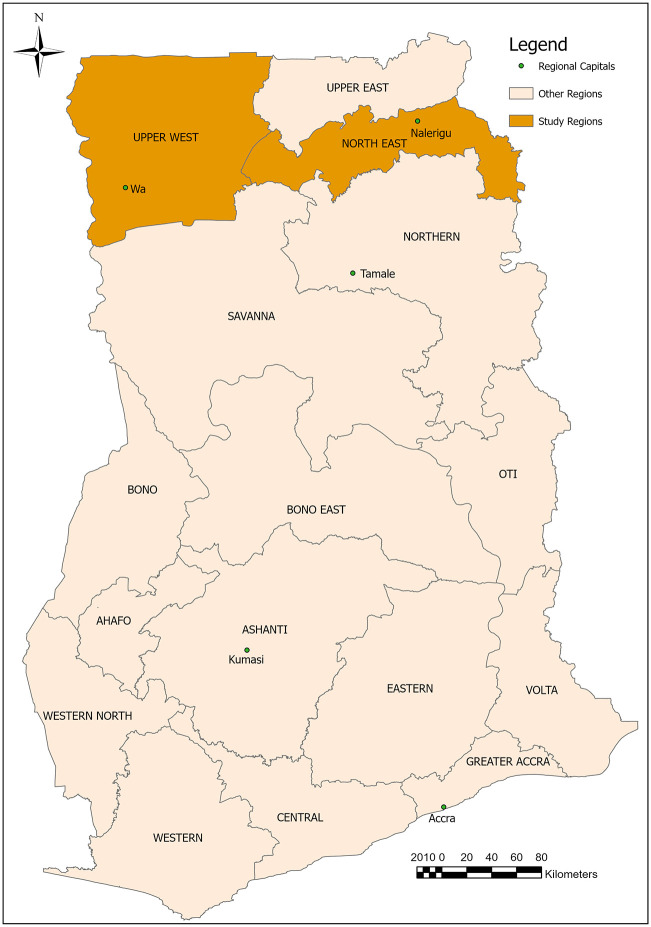
A map of Ghana showing the study regions (ArcGIS Pro software version 3.3.1, ESRI Inc.).

A retrospective study design was used to examine the presentation and clinical management of snakebite patients over a five-year period in two hospitals in the Upper West and North East regions of Ghana (Fig 1). Selection of the study regions is based on data retrieved from the District Health Information Management System (DHIMS2) which estimates the hospital attendance rate on account of snakebite in Upper West and North East regions as 167 and 60 persons per 100,000 population per year respectively. These are above the estimated national average of 35 persons per 100,000 population per year [6].

### Study population

Both regions are characterised by grassland with scattered drought-resistant trees, providing a habitat for similar snake species. About 2.9% and 2.2% of the total population of Ghana reside in the Upper West and North East regions, respectively [29]. Both regions are predominantly agrarian with most of the population engaged in rudimentary subsistence farming activities. Residential structures in these regions are mainly made of materials such as mud, reed, straw and thatch. The aforementioned occupation and residential structures in both regions have been found to contribute to an increased risk of snakebite [30,31].

The surveyed hospitals are located in their regional capitals and are among the major health facilities in the two regions, both offering specialist services including internal medicine, surgery, physiotherapy, obstetrics/gynaecology and paediatrics services. The Wa Municipal Hospital (WMH), which is located in the Wa Central Municipality of the Upper West region, is a public hospital with a capacity of about 210 beds and a medical staff of over 410. Until 2019, it served as the Upper West regional referral hospital and treats about 72,000 patients annually. The Baptist Medical Centre (BMC) is a faith-based healthcare provider that serves as the regional referral hospital in the North East region. The hospital has a bed capacity of about 310 and a workforce of about 340 healthcare workers. Located in Nalerigu in the East Mamprusi district, the BMC is the largest health facility in the North East region and attends to over 110,000 patients annually. Due to the specialist services provided in both the WMH and the BMC, they have been the preferred destination for patients as well as referral centres for peripheral health facilities for the management of snakebite in both regions.

### Data collection

All records of patients reporting on account of snakebite and diagnosed based on the national Standard Treatment Guidelines (STG) [32] and the International Classification of Diseases codes from 2016 to 2020 inclusive in WMH and BMC were eligible for inclusion in the study. The identity numbers of folders containing patients’ medical records were retrieved from the electronic record systems by the Information Officer of the WMH and the Information Technology Officer of the BMC and the hard-copy folders and consulting room registers retrieved by the records unit of both hospitals. All patients whose medical records were not available or missing all the following variables were excluded: age, date the bite occurred, anatomical site of the bite, and symptoms upon admission. The data was collected in August 2019 and August 2022 at the BMC and between February and March 2021 at the WMH. On average, eight trained healthcare personnel affiliated to the hospitals conducted the data entry process at each hospital. The Research Electronic Data Capture (REDCap) tools (version 12.5.6) hosted by the Global Health and Infectious Diseases Research group at the KCCR, Ghana was used for the data collection process [33,34].

### Data analysis

Demographic characteristics and patients’ clinical data were analysed using relative frequencies to describe the presenting clinical signs and symptoms, treatments received and the resulting health outcomes. Seasonal and monthly variations were assessed to identify periods during which the risk of snakebite envenoming is elevated. The seasons were defined as rainy (March to September) and dry (October to February) [35]. A nonparametric trend analysis using LOESS (Locally Estimated Scatterplot Smoothing) curves for the number of patients reporting on account of snakebite over the period under review was conducted. This technique uses local weighted regression to fit a smooth curve through the time series. The time of hospital presentation relative to the time of the bite, duration of hospitalisation, medications administered, medical complications, and health outcomes were compared between the two hospitals using relative frequency and relative risk. Patients with systolic blood pressure (SBP) < 90 mmHg were identified as having low SBP based on the national Standard Treatment Guidelines (STG) for shock by the Ghana Ministry of Health [32]. All clinical signs and symptoms not indicated in the patient’s record book were considered not present at the time of clinical examination. All other missing data were assumed to be missing completely at random. The pairwise deletion approach was used to handle missing data during the data analysis process. IBM SPSS Statistical software version 28 for Windows was used to conduct the data analysis.

## Results

A total of 2,163 and 1,661 folder identity numbers of patients reporting on account of snakebite were retrieved from the record systems of the Wa Municipal Hospital (WMH) and the Baptist Medical Centre (BMC), respectively. Based on the cumulative total population of about 6.96 million in both regions over the study period and a total of 3,824 patients reporting on account of snakebite over the period in both hospitals, the annual hospital attendance rate on account of snakebite is estimated at 55 persons per 100,000 population per year in both health facilities. Due to instances of in-patients absconding with their folders to evade paying hospital bills prior to discharge, misplaced and missing folder details, and the random destruction of folders by insects and rodents, a total of 1,270 and 1,414 physical folders were retrieved and included in the study from the WMH and the BMC, respectively (Table 1).

**Table 1:** Demographic characteristics of patients.

Variable	Baptist Medical Centre (n = 1,414)	Wa Municipal Hospital (n = 1,270)	Total (N = 2,684)
Bites per year (n %)			
2016	316 (22.3)	287 (22.6)	603 (22.5)
2017	287 (20.3)	319 (25.1)	606 (22.6)
2018	344 (24.3)	279 (22.0)	623 (23.2)
2019	287 (20.3)	228 (17.9)	515 (19.2)
2020	180 (12.7)	152 (12.0)	332 (12.4)
Not indicated	0 (0.0)	5 (0.4)	5 (0.2)
Gender (n %)			
Male	932 (65.9)	805 (63.4)	1737 (64.7)
Female	482 (34.1)	465 (36.6)	947 (35.3)
Age in years			
Median (IQR)	20.0 (12.0 – 32.0)	21 (12.8 – 35.0)	20.0 (12.0 – 33.3)
Anatomical site of bite (n %)			
Head and Neck	8 (0.6)	1 (0.1)	9 (0.3)
Trunk	8 (0.6)	5 (0.4)	13 (0.5)
Upper limbs	319 (22.6)	209 (16.5)	528 (19.7)
Lower limbs	939 (66.4)	965 (76.0)	1904 (70.9)
Not indicated	140 (9.9)	90 (7.1)	230 (8.6)
Time period of bite (n %)			
Morning	181 (12.8)	165 (13.0)	346 (12.9)
Afternoon	48 (3.4)	123 (9.7)	171 (6.4)
Evening	122 (8.6)	233 (18.3)	355 (13.2)
Night	58 (4.1)	98 (7.7)	156 (5.8)
Not indicated	1005 (71.1)	651 (51.3)	1656 (61.7)
Season of bite (n %)			
Rainy	1038 (73.4)	783 (61.7)	1821 (67.8)
Dry	376 (26.6)	477 (37.6)	853 (31.8)
Not indicated	0 (0.0)	10 (0.8)	10 (0.4)

**Fig 2:**
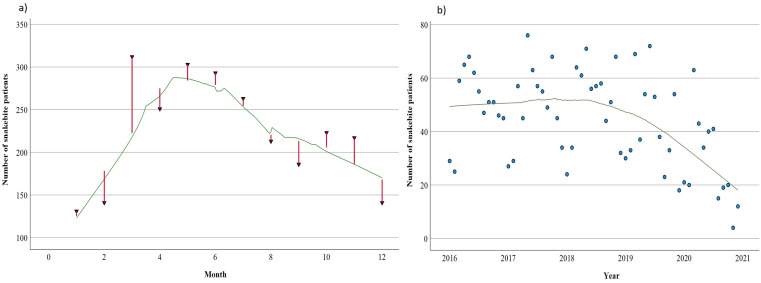
LOESS smooth curves of patients reporting to the hospitals on account of snakebite from 2016 to 2020. **a)** Monthly trend with spikes in number of reported patients **b)** Annual trend of reported patients from January 2016 to December 2020.

In total, the records of 2,684 snakebite patients who presented to both health facilities between 2016 and 2020 were reviewed. Male patients accounted for 64.7%. The median (IQR) age of the patients at the time of bite was 20 (12.0 – 33.3) years. The LOESS smooth curves show a peak in snakebites from March to July (Fig 2). This correlates with the rainy season. The annual trend shows fairly constant case presentations until 2019, when the decline in case presentations started. The suspected snake species responsible for the bites were not indicated in the patients’ medical records.

Of the 2,684 patients, 11.7% were referred from peripheral health facilities (Table 2). 2,449 (91.2%) of the reporting patients were admitted to the ward after triage at the emergency departments. About 72% (1,923) of all the patients reported to the hospital on the same day the bite occurred. Within this group who reported on the same day of the bite, 70.6% reported directly to either of the study hospitals, 12.2% were referred from other health facilities on the same day the bite occurred and 17.3% did not have data on their referral status. Time to presentation to the hospital was recorded in days. Only 1.8% reported later than 5 days after the snakebite. The longest delay before reporting to a health facility was 22 days.

**Table 2:** Hospitalisation and clinical features.

Variable	Baptist medical centre (n=1,414)	Wa municipal hospital (n=1,270)	Total (N=2,684)
**Referred from other health facilities (n %)**			
Yes	28 (2.0)	286 (22.5)	314 (11.7)
No	981 (69.4)	813 (64.0)	1794 (66.8)
Not indicated	405 (28.6)	171 (13.5)	576 (21.5)
**Admission status (n %)**			
Admitted into the hospital ward	1233 (87.2)	1216 (95.7)	2449 (91.2)
Emergency department only	181 (12.8)	54 (4.3)	235 (8.8)
**Length of hospitalisation Median number of days (IQR)**	3.0 (2.0 – 4.0)	2.0 (1.0 – 3.0)	2.0 (2.0 – 4.0)
**Systolic blood pressure (n %)**			
Low (< 90 mmHg)	16 (1.1)	133 (10.5)	149 (5.6)
Normal/High (≥90 mmHg)	908 (64.2)	828 (65.2)	1736 (64.6)
Not indicated	490 (34.7)	309 (24.3)	799 (29.8)
Presenting clinical signs* (n %)			
Bleeding	239 (16.9)	345 (27.2)	584 (21.8)
Pallor	100 (7.1)	52 (4.1)	152(5.7)
Swelling	554 (39.2)	895 (70.5)	1449 (54.0)
Severe pain	328 (23.2)	603 (47.5)	931 (34.7)
Lethargy	13 (0.9)	27 (2.1)	40 (1.5)
Other signs	15 (1.1)	12 (0.9)	27 (1.0)

* Some patients had multiple clinical signs.

Only 2% of the records had data on first aid interventions administered before reporting to the hospital. These include visiting a traditional healer for treatment, making incisions at the bite site, applying herbs or blackstone (believed to have venom absorbing and healing effects) to the bite site, and using tourniquets. Patients who reported to the WMH in the Upper West region had a higher relative risk (RR, 1.10; 95% CI, 1.07–1.12) of being admitted compared to patients reporting to the BMC in the North East region. Swelling, severe pain and bleeding were the most common clinical signs upon presentation. Although 584 patients presented with some form of bleeding, only 9.6% had a low SBP. The site and extent of bleeding were not specified in the medical records.

**Fig 3:**
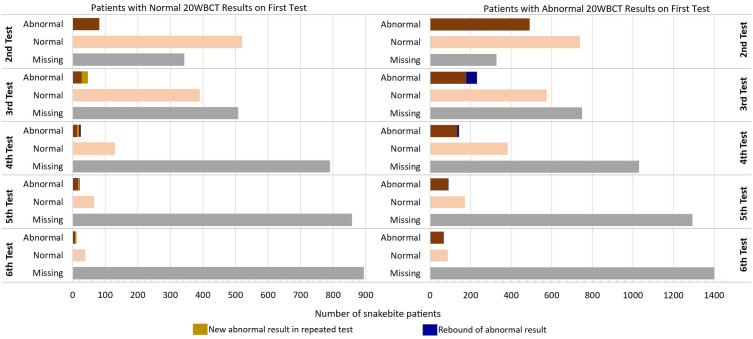
Repeated 20-minute whole blood clotting test results categorised based on the results of the first clotting test.

At least one 20WBCT was conducted for 96.2% (2,581/2,684) of the patients. However, results were available for 2,526 patients. 1,670 (66.1%) of all the patients with 20WBCT results indicated had at least one abnormal clotting result; 93.2% had an abnormal clotting result on their first test while 6.8% had normal clotting results on the first test but recorded abnormal results on subsequent tests (Fig 3). The specific time for repeated clotting test is not often recorded. However, the general practice is to repeat the test between 4–6 hours.

Within the total population with at least one abnormal 20WBCT result, 24.1% (403/1,670) reported with bleeding and 23.1% (93/403) of this subgroup received blood transfusion. Among the patients who recorded normal 20WBCT results, 18% (154/856) reported with some form of bleeding, with 18.8% (29/154) of the patients in this subgroup receiving blood transfusion.

**Fig 4:**
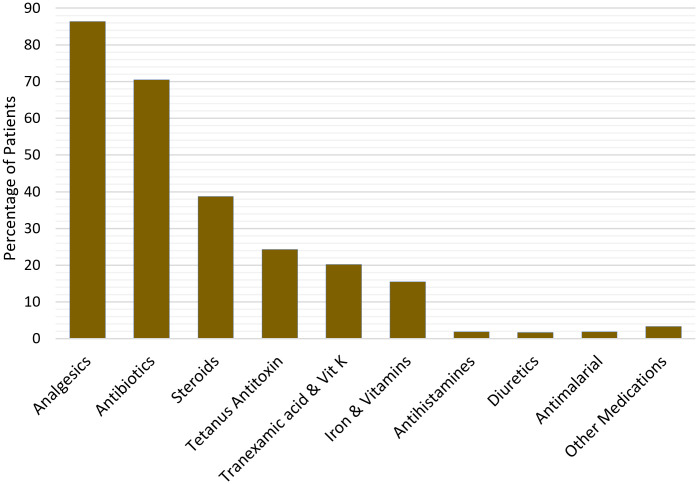
Classification of administered medications.

Of the 1,670 who had at least one abnormal clotting test result, 96.6% (1,614) were treated with antivenom compared to 64.3% (550/856) of those who did not record any abnormal clotting results. The median (IQR) number of antivenom vials received per patient was 2 (1–3) vials. However, 33 patients received 10 or more vials for their treatment. The specific type of antivenom administered was not recorded. A total of 73 (2.7%) patients experienced a rebound of abnormal clotting results and were retreated with antivenom, after ceasing treatment when normal clotting was achieved from initial treatment. Analgesics were the main ancillary medications administered for the management of snakebite induced pain (Fig 4). Paracetamol was administered to 73.3%, opioids to 13.9% and non-steroidal anti-inflammatory drugs (NSAIDS) to 12.3%. Steroids were given to 38.4% of the patients. Hydrocortisone accounted for over 99% of the steroids administrated, adjuvant to antivenom. Four patients received adrenaline but with unclear clinical indication.

**Fig 5:**
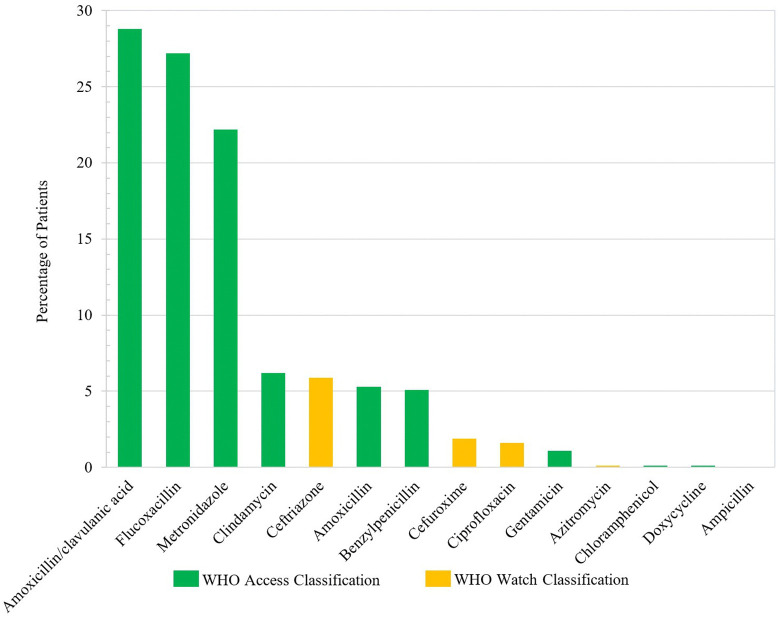
Antibiotics administered as part of snakebite treatment.

Antibiotics were administered to 70.5% of the patients. Among whom, 59.2% received one antibiotic agent, 32.3% received two different agents and 8.5% received three to five different agents of antibiotics in the course of treatment. Amoxicillin with clavulanic acid, flucloxacillin and metronidazole were the most commonly administered antibiotics (Fig 5). Four out of the 14 administered antibiotics are on the World Health Organisation’s (WHO) watch list based on the Access, Watch and Reserve (AWaRE) classification.

Severe complications that were recorded included compartment syndrome (two patients), intraocular haemorrhage (two patients) and submandibular hematoma (two patients). Gangrene was diagnosed in eight patients and necrosis in four patients. Respiratory distress with dysphagia, hypoxia and seizures were observed in three patients. Cellulitis was reported for 28.8% of all patients without further description of the clinical signs. Anaemia was reported for 4.5% of the patients.

The total recorded case-fatality rate was 1.9% (Table 3). The Upper West recorded a case-fatality rate of 2.8% compared to 1.1% in the North East. However, in the North East region, 2.3% of the patients were referred from the BMC to other tertiary health facilities for advanced care and 0.1% discharged against medical advice. No referrals for advanced care were made from the WMH but 6% of patients absconded or were discharged against medical advice. The health outcomes of these patients are unknown. Furthermore, 22.6% of the patients reporting in the Upper West region were referred by other peripheral health facilities to the WMH compared to the 2% referrals received at the BMC in the North East region (Table 2). Patients who recorded abnormal clotting results but were not treated with antivenom were 56 in total. Out of this number, 16.1% reported with some form of bleeding, 48.2% with swelling and 30.4% with severe pain. Two patients within this group died and 9 were referred for advanced care.

**Table 3:** Treatment and health outcomes.

Variable	Baptist medical centre (n=1,414)	Wa municipal hospital (n=1,270)	Total (N=2,684)
**Antivenom administration (n %)**			
Antivenom administered	1184 (83.7)	1078 (84.9)	2262 (84.3)
Antivenom not administered	230 (16.3)	169 (13.3)	399 (14.9)
Missing	0 (0.0)	23 (1.8)	23 (0.9)
**Number of antivenom vials**			
Median (IQR)	2.0 (2.0 – 3.0)	2.0 (1.0 – 2.0)	2.0 (1.0 – 3.0)
**Health outcome (n %)**			
Recovered	1350 (95.5)	1143 (90.0)	2493 (92.9)
Amputation	3 (0.2)	1 (0.1)	4 (0.1)
Referred for advanced care	33 (2.3)	3 (0.2)	36 (1.3)
Absconded/discharged on request	2 (0.1)	76 (6.0)	78 (2.9)
Died	15 (1.1)	35 (2.8)	50 (1.9)
Not indicated	11 (0.8)	12 (0.9)	23 (0.9)

The median age of the patients who died is 31 (15 – 45) years. The majority of them were male patients (62%). Four (8%) of the patients with fatal outcomes were referred from other health facilities to the WMH.

## Discussion

The hospital attendance rate on account of snakebite is estimated at 55 persons per 100,000 population per year in the WMH and the BMC. The main clinical sign and symptoms exhibited by snakebite patients in these regions are clotting disorders, swelling and severe pain which are consistent with haemotoxins envenoming from species such as *Echis ocellatus* and *Bitis arietans*. The majority of the patients in the study were treated with antivenom. The main venom-induced medical complication recorded are compartment syndrome, intraocular haemorrhage, submandibular hematoma and amputation. The total facility-based fatality rate over the five-year period under review was 1.9%.

Existing evidence suggest snakebite envenoming exerts a high burden on the health system in northern Ghana [5,18,36], where the average doctor-to-patient ratio was estimated at about 1:14,200 in 2020 [37]. Our results show a similar trend with the two health facilities each handling an average annual case load of about 765 snakebite patients. In 2019 a new regional hospital was commissioned the Upper West regions. This may have accounted for the decrease in case presentations to the WMH. Diagnosis and treatment of snakebite patients was guided by the 20WBCT and presenting clinical signs and symptoms. This approach has been documented to be practiced in many resource-limited settings with poor diagnostic capabilities [12–14]. Although there was limited data on the type and brand of antivenom administered, only three polyvalent antivenom brands – PANAF-Premium (Pan Africa), AFRIVEN (Snake Venom Antiserum (African 10) I.H.S and ALS-SVAS (Snake Venom Anti Serum) (African)) – are listed on the Ghana Food and Drugs Authority Products Register [38]. Nevertheless, there are other brands and types of antivenoms in the open market, including ECHIVEN (Snake Venom Antiserum (ECHIS) I.H.S) a monovalent antivenom targeting Echis species as well as other antivenoms.

Generally, the length of hospitalisation was short in comparison with other studies in Ghana in which average length of hospitalisation ranged from 2.7 to 4.7 days [19,27,28,39]. The short length of hospitalisation may be linked to early presentation and most envenoming being haemotoxic and thus, may require shorter hospital stays compared to cytotoxic and neurotoxic bites from species such as *Bitis arietans, Naja nigricollis, Dendroaspis viridis* or *Dendroaspis polylepis.* Patients bitten by these species may require more extensive treatment and rehabilitation such as surgical procedures, wound care, respiratory support and physiotherapy [40,41]. Similar fatality rate was recorded in comparison with studies conducted in mid-and northern Ghana and north-eastern Nigeria in which recorded fatality rates ranged between zero to 1.6% [19,27,28,39,42]. However, the case-fatality rate in the Nigerian study increased more than two folds during the months of antivenom stock-out [42].

Repeating the 20WBCT is essential in averting complications of coagulopathic envenoming by detecting persistent or rebound coagulopathy [43,44]. In our study population, delayed and/or rebound coagulopathy was detected in more than 6% of the patients.

Among the patients who were not treated with antivenom, 56 (14%) recorded abnormal clotting results. Reasons for the non-administration of antivenom include stock-outs and patients’ inability to afford or access antivenom from the open market, patients absconding or discharged against medical advice or patients dying before antivenom could be administered. On the contrary, 648 (63.9%) of the patients with normal clotting results were treated with antivenom. In these patients, signs of systemic envenomation or severe local swelling are likely to be the primary reason for antivenom therapy [45]. Although a significant proportion of the patients in this study reported with swelling or bleeding, the extent and severity were not documented. It is therefore plausible that patients with dry bites or non-venomous bite may have been treated with antivenom. With the 20WBCT having a sensitivity of 82–89% and a specificity of 82–98%, some patients with coagulopathic envenoming may have gone undetected [40]. The 20WBCT may therefore be insufficient to identify all patients with coagulopathic envenoming in need of antivenom treatment. Although the national STG recommends 5–10 vials of antivenom depending on the severity of envenoming and repeating antivenom administration as required based on the patient’s response to treatment, we ascertained that clinicians often administer an average of two vials and repeat after about six hours depending on the severity or the persistence of clinical signs of envenoming and results of the repeated 20WBCT. This conservative approach is adopted due to the limited supply of antivenom in these high-risk settings. This highlights the need to ensure adequate stocking and judicious use of appropriate antivenom.

Aside antivenoms, ancillary medications are essential in treating other bite or venom induced signs and symptoms. Even though tetanus as a complication of snakebite is rare, the Ghanaian STG as well as the WHO guidelines recommends a prophylactic dose of anti-tetanus therapy for all snakebite patients [5,32]. However, less than 25% of the patients in this study population received anti-tetanus therapy. Paracetamol was the main medication used for pain management, although NSAIDs were administered to a considerable number of patients despite being identified as a relative contraindication particularly in patients with haemotoxic envenoming [46–48]. It is unclear whether adequate pain assessment was conducted, and management undertaken according to the WHO analgesic ladder [49]. Similarly, the national treatment guideline and prevailing evidence do not support the administration of antifibrinolytic agents, such as tranexamic acid or vitamin K for snakebite management [50–52].

Steroids, particularly hydrocortisone, are often administered by clinicians in our study setting. In the WHO guidelines, there is no indication for the use of steroids [5] and clinical trials have also showed no effect against a biphasic course of anaphylaxis in SBE patients [53–55]. In this study, only four patients were treated with adrenaline, but the exact indications are unclear. These four patients were among the deceased. The adrenaline may have been used as part of resuscitation efforts although anaphylactic shock was not recorded in their medical records. Training and making research evidence readily available to clinicians via updated STGs could improve clinical decision-making and help alleviate the socioeconomic and health system burden of SBE.

Even though most studies do not support the treatment of SBE patients with antibiotics [56–62], the majority of the patients in this study were treated with antibiotics. Four of the administered antibiotics are on the WHO AWaRE classification list, which is aimed at emphasizing the significance of appropriate use while also taking into account the impact of various antibiotics and antibiotic classes on antimicrobial resistance [63]. In the national STG, only amoxicillin has been recommended for the prevention of secondary infections. In the regional guideline for Africa, it is recommended that treatment with antibiotics should be delayed until definite signs of infection arise or when the bite site has been interfered with. Limited knowledge on the pathophysiology of infections such as cellulitis [64,65] may have contributed to the frequent administration of antibiotics, considering the high percentage of patients recorded to have developed cellulitis. Cellulitis was often diagnosed in less than 48 hours upon arrival to the health facility; a time frame within which a true bacterial infection is unlikely to establish itself in skin tissue. This suggests swelling or erythema after the bite was likely recorded as cellulitis in many cases. With limited indications documented in the medical records, the use of antibiotics, primarily as prophylaxis, demonstrates poor adherence to treatment guidelines.

There are limitations to this study given its retrospective nature, including that no data on snake species, specific antivenom utilised or systematic data on outcomes were available in the medical records. The misplacement or unavailability of patient’s medical records occurred at random. The non-standardisation of record-keeping between the health facilities and among clinicians may have also introduced procedural bias. Hence, the findings should be interpreted with caution. Nevertheless, the same data collection approach was employed in both health facilities to ensure uniformity in the structure and data collection process. Subsequently, all health facilities in the country have adopted the Lightwave Health Information Management System (LHIMS); an electronic medical information management platform that aims to standardise and improve medical data collection and storage.

## Conclusion

With an estimated annual hospital attendance rate on account of snakebite being 55 persons per 100,000 population per year, snakebite is a major public health challenge and a burden to the Wa Municipal Hospital and the Baptist Medical Centre. Haemotoxic envenoming is the main clinical presentation but low mortality rates are recorded with a high number of patients treated with antivenom. More evidence on the clinical management of snakebite envenoming and better use of available evidence on ancillary care is needed to guide decisions.

## Supporting information

S1 Appendix West African Carpet Viper (*Echis ocellatus*). (DOCX)
