# Supplementary material for: Clinical presentation and management of snakebite envenoming in northern Ghana
Source: PLoS Negl Trop Dis. 2025 Dec 12;19(12):e0013820. doi: 10.1371/journal.pntd.0013820 (PMC12716685; doi:10.1371/journal.pntd.0013820)
Supplement: S1 Appendix — (DOCX) [file pntd.0013820.s001.docx]

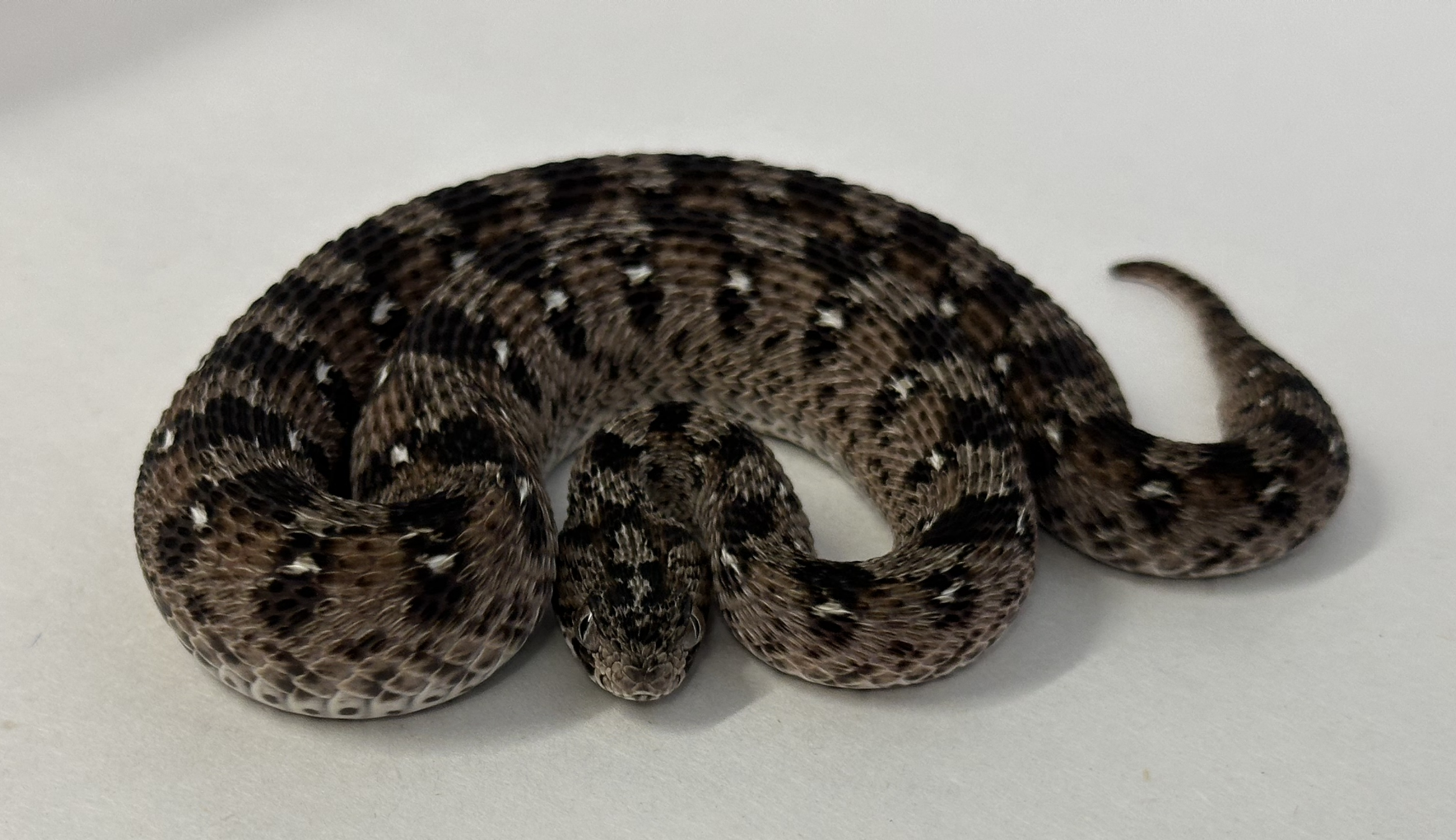


Photo Credit: Paul D. Rowley, Senior Herpetologist, Centre for Snakebite Research & Interventions, Liverpool School of Tropical Medicine, UK
